# Effects of lithium on locomotor activity and circadian rhythm of honey bees

**DOI:** 10.1038/s41598-023-46777-7

**Published:** 2023-11-13

**Authors:** Babur Erdem, Okan Can Arslan, Sedat Sevin, Ayse Gul Gozen, Jose L. Agosto-Rivera, Tugrul Giray, Hande Alemdar

**Affiliations:** 1 Department of Biological Sciences, Middle East Technical University, Ankara, Turkey; 2 Center for Robotics and Artificial Intelligence (ROMER), Middle East Technical University, Ankara, Turkey; 3 Department of Pharmacology and Toxicology, Faculty of Veterinary Medicine, Ankara University, Ankara, Turkey; 4 Department of Biology, University of Puerto Rico, Rio Piedras, Puerto Rico; 5 Department of Computer Engineering, Middle East Technical University, Ankara, Turkey

**Keywords:** Neuroscience, Circadian rhythms and sleep

## Abstract

Lithium has been considered a potential acaricidal agent against the honey bee (*Apis mellifera*) parasite *Varroa*. It is known that lithium suppresses elevated activity and regulates circadian rhythms and light response when administered to humans as a primary therapeutic chemical for bipolar disorder and to other bipolar syndrome model organisms, given the crucial role of timing in the bee's foraging activity and the alternating sunlight vs dark colony environment bees are exposed, we explored the influence of lithium on locomotor activity (LMA) and circadian rhythm of honey bees. We conducted acute and chronic lithium administration experiments, altering light conditions and lithium doses to assess LMA and circadian rhythm changes. We fed bees one time 10 μl sucrose solution with 0, 50, 150, and 450 mM LiCl in the acute application experiment and 0, 1, 5, and 10 mmol/kg LiCl ad libitum in bee candy in the chronic application experiment. Both acute and chronic lithium treatments significantly decreased the induced LMA under constant light. Chronic lithium treatment disrupted circadian rhythmicity in constant darkness. The circadian period was lengthened by lithium treatment under constant light. We discuss the results in the context of *Varroa* control and lithium's effect on bipolar disorder.

## Introduction

*Varroa* is a devastating parasite of honey bees. The lethal effect of lithium on *Varroa* was surprisingly discovered by a research group while attempting to find an iRNA-based treatment for *Varroa* control^1^. The lethal effect of lithium on *Varroa* was considered promising since when bees fed on a 10 µl sucrose solution with a concentration of at least 25 mM LiCl, a 24-h application had no effect on bee survival. However, chronic feeding results in reduced honey bee lifespan^1^. Lithium is not yet used as an anti-*Varroa* agent, and further studies on lithium's effect on honey bees are warranted.

Studies on lithium's effect on bee health demonstrated positive and negative impacts. Lithium treatment reduced viral load and mitigated oxidative stress by altering gene expression^2^. Other studies reported toxicity of the residual lithium on honey bee colonies^3,4^ and significant brood damage^5^. Also, lithium applied to honey bee colonies was found to accumulate in all compartments and members of the hive^6^. It is important then, to consider the sublethal effects of lithium, for instance, on honey bee behavior. Currently, few reports are available on the behavioral effects of lithium applications on honey bees. One behavioral study investigated lithium-induced malaise behavior in honey bees: injection or ingestion of LiCl decreased walking and increased stillness^7^. Only one study examined the acute effect of lithium salts on locomotor activity (LMA), demonstrating differences based on the dose and type of lithium salt used^8^.

Studies on the behavioral effects of lithium on bees may also be informative for lithium’s actions as a drug. In medicine, lithium is known as the first-line treatment for bipolar disorder because of its mood-stabilizing activity and low toxicity^9^. In our study, we examined the effect of lithium on LMA and honey bees' circadian rhythm. We hypothesized that the lithium effect on LMA and circadian rhythms will depend on the light environment and reflect the moderating effect of lithium on bipolar disorder symptoms.

Repetitive shifts between unusual mood levels, such as depression and mania, characterize bipolar disorder. Irritable mood, abnormally and persistently above-average activity or energy, and reduced need for sleep are observed in the manic episode^10^. In addition to spikes of activity, circadian rhythm abnormalities accompany bipolar disorder^11^. Lithium stabilizes activity levels in individuals with bipolar disorder. Furthermore, lithium treatment decelerates the fast circadian clock observed in individuals with bipolar disorder^12,13^. However, lithium’s action mechanism remains only partially understood, and the studies investigating lithium’s effect on LMA and circadian rhythm provide limited answers^14^. That limitation is partly due to the lack of suitable animal models for bipolar disorder^9^.

At times, honey bees and fruit flies serve as insect models for human disorders. Multiple models, due to nuances of molecular, neural, and developmental substrates, result in improved fundamental understanding. For instance, in the case of the circadian clock, the honey bee insect model has a circadian rhythm ontogeny that is similar to the humans. Post-embryonic development of the circadian clock has been observed in bees, as in human infants^15^. In addition, bees have a protein called pteropsin. This protein is more closely related to vertebrate opsins than to invertebrate opsins. Vertebrate opsins are involved in circadian rhythm regulation^16^. Also, bees lack *Drosophila* cryptochrome; instead, bees encode an ortholog of the two mammalian cryptochromes^16^. These studies suggest bees are more similar to vertebrates than other insects in terms of ontogeny and molecular mechanisms of circadian rhythms.

Until now, we did not have studies on the effects of lithium on the circadian rhythms of honey bees. For lithium, research on the fly model indicated lengthening of the circadian period, increasing longevity, and positive effects on locomotion via *Shaggy* (*Sgg*, insect orthologue of vertebrate *Gsk-3*) inhibition ^17^. According to a study conducted on fruit flies, concentrations of 20 mM and 30 mM LiCl significantly extended the period of circadian rhythm to 24.02 h and 24.4 h, respectively, when compared to the control group, which maintained a period of 23.7 h. Additionally, lithium increased arrhythmicity, particularly at doses above 5 mM, and the arrhythmicity ratio exceeded 50% at 60 mM application^14^. Another study on fruit flies indicated that the period of the circadian rhythm increased *Sgg* loss of function mutants^18^. In a study examining longevity and locomotion, the fruit fly life expectancy increased with *Sgg* inhibition following the application of 1–25 mM LiCl. In addition, the treatment ameliorated the decrease in aging-dependent LMA^19^. It is known that the disruption of circadian rhythm is frequently observed in humans with bipolar disorder^20^. Bipolar disorder-driven sleep latency alterations and a reversal in the sleep/wake period were observed in humans^21^. As in fruit flies, circadian rhythm phase anomalies in humans were shown to be modified with lithium treatment acting as an *Sgg* inhibitor^22^. It is important to contrast the effects of lithium on flies to the effects of lithium on bees.

Honey bees have already provided valuable insights into various complex behaviors with parallels to human behavior. For instance, cocaine affected the reward perception and withdrawal-like responses, reducing the learning ability of honey bees during the withdrawal period as in humans^23^. In another example, honey bees were successfully employed as an ethanol-abuse model^24^. Dopamine, a biological amine common to humans and bees, was found to affect learning and motivation in honey bees^25^. A remarkable study showed mechanistically relevant behavioral similarities between humans and honey bees. The study indicated that socially unresponsive worker bees had similar gene expression patterns for the homologous versions of the autism-related genes in humans^26^. These diverse neural genetic substrates of behavior make the honey bee a good model animal for lithium research on circadian rhythm.

We argue honey bees provide an informative and relevant insect model for investigating lithium's effects on LMA, circadian rhythm, and behavior. The bee model is informative because circadian rhythm regulation has become a prominent research focus within the field of bee studies. These studies show honey bee circadian rhythms and locomotion are sensitive to light regimes and other time givers^15,16,27^. The model is relevant because circadian regulation of bee activity is important for foraging and pollination. It is known that foraging activity is modified based on the time flowers provide nectar during the day^28^, resulting in the maximization of collected resources. Daylight is also significant for accurately navigating to and from nectar and pollen resources and communicating their location to nest mates using the sun-compass orientation that is tuned to the intrinsic circadian clock of the bee^29^. Lastly, the circadian rhythm of honey bees is susceptible to chemical treatments^30,31^ bees face in their environment and due to human activities.

It is known that the increase in excessive activity, an important component of mania, which is the most prominent symptom of bipolar disorder, is balanced by lithium^9,10^. In the honey bee model, exposure to light leads to higher activity^27^. We hypothesize that lithium will inhibit induced activity also in the honey bee model. This suppression of induced activity hypothesis will be supported if lithium reduces the elevated LMA induced by light in honey bees. Secondly, previous studies have shown that lithium treatment changes circadian rhythm parameters such as period length^14,32^, rhythmicity, and light sensitivity of the clock^33–39^. We hypothesize that lithium will affect the light input to the biological clock in the honey bee also. This regulation of circadian activity hypothesis will be supported if lithium alters the parameters of circadian rhythm under chronic conditions in a light-dependent manner in the honey bee model.

In order to test our hypotheses, we manipulated light conditions in acute and chronic lithium exposure experiments to investigate light-induced LMA and circadian rhythm changes. We discuss the results in relation to suppression of induced activity and regulation of circadian rhythmicity hypotheses and compare the results to data from bipolar disorder models and humans.

## Results

### Acute effects of lithium on locomotor activity

In acute LMA trials, we used three doses of LiCl (low, medium, and high) and one dose of NaCl with a control group. The low, medium, and high doses of LiCl were 50 mM, 150 mM, and 450 mM, respectively. The concentration of the NaCl applied in the acute LMA experiment was 450 mM.

The LMA measurements did not follow the normal distribution (Shapiro–Wilk test, *p* < 0.05). A non-parametric Kruskal–Wallis test was used to compare the treatment groups for light and dark environment experiments. In addition, descriptive statistics are found in Supplementary Table S1.

**Figure 1:**
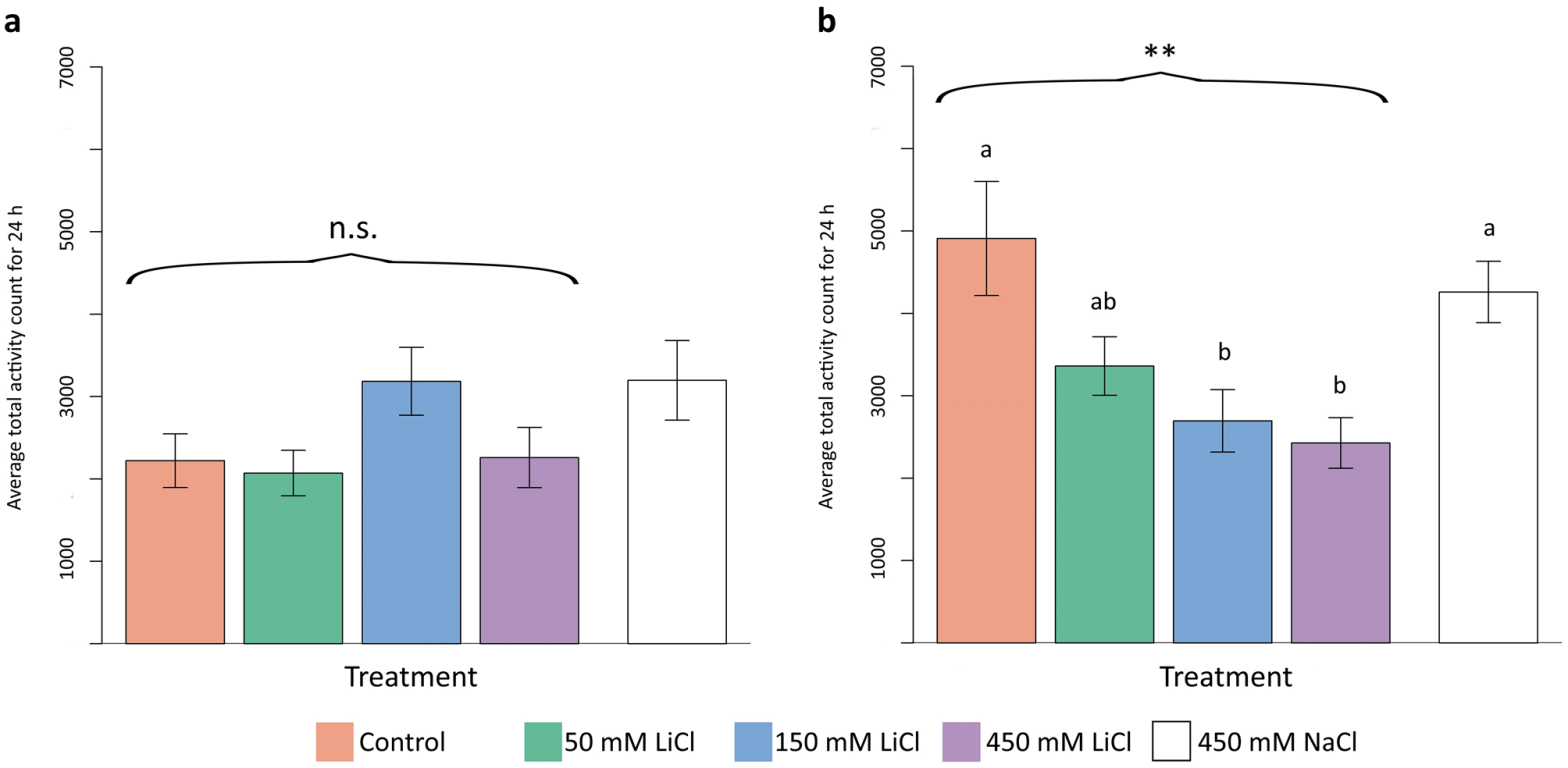
The figure illustrates the comparisons of LMA in response to acute lithium administration experiments conducted in both dark (**a**) and light (**b**) environments. In cases where a significant difference was detected among the groups according to the Kruskal–Wallis test followed by a post-hoc Dunn test, letters were added to the bars. When the same letters are present, it signifies that there is no statistically significant difference between the groups. Curly brackets are employed to convey the results of regression analysis. Asterisks denote the statistical significance level with the following thresholds: *p ≤ 0.05, **p ≤ 0.01, ***p ≤ 0.001, and n.s. indicating that it is not significant. The data is presented as the mean of the total activity count with ± standard error.

First, we compared the LMA differences in the dark environment experiment. There were no significant differences among groups (Kruskal–Wallis test: *H* (4) = 9.11, *p* = 0.060; Fig. 1A).

Next, we compared the LMA of the groups in the light environment, and we found significant differences (Kruskal–Wallis test: *H* (4) = 16.86, *p* < 0.001; Fig. 1B). A post-hoc Dunn test indicated that the control group differed from the medium (*p* = 0.004) and high-dose groups (*p* < 0.001); similarly, the NaCl group varied from the medium (*p* = 0.004) and high-dose groups (*p* < 0.001), and there was no difference observed between control and NaCl groups (*p* = 0.486).

Then, we investigated the relationship between doses of LiCl (the NaCl group was excluded) and LMA using regression analysis. In dark environment experiments, there was not a significant association between dosages of lithium and activity (*R*^*2*^ = 0.0001, *F* (1, 92) = 0.01, *p* = 0.916) (Fig. 1A). However, the regression analysis indicated a significant association between dosages of lithium and activity (*R*^*2*^ = 0.1, *F* (1, 84) = 9.18, *p* = 0.003) in the light environment experiments (Fig. 1B). As a result, increasing the dosage lowered the activity in the light environment but did not affect activity in the dark environment.

In addition, a chi-squared test was applied to find any difference across all groups, including both dark and light environment experiments regarding the mortality ratio in the groups. The test indicated a difference across the groups (*χ*^*2*^ (9, 263) = 66.40, *p* < 0.001). The high-dose group in the dark environment had a higher death ratio (0.48 in the dark condition and 0.17 in the light condition). However, this higher death ratio was an outlier when the mortality ratios of all experimental groups in both dark and light conditions were compared (Supplementary Figs. S1, S2). It has been difficult to causally explain high mortality only in this one group.

### Chronic effects of lithium on locomotor activity and circadian rhythm

In chronic LMA experiments, we compared three doses: low (1 mmol/kg), medium (5 mmol/kg), and high (10 mmol/kg), and a control group (no lithium administered).

We first analyzed the effect of lithium on survival. According to the survival analysis, the high-dose group had a lower survival rate (0.34) than other groups (control: 0.63, low: 0.56, medium: 0.63) on the 15th day. The difference between the groups was indicated by a log-rank test (*χ*^2^ = 7.9, *p* = 0.05) (Supplementary Fig. S3). Still, according to pairwise comparisons, none of the lithium groups differs from the control (*p* > 0.05) (Supplementary Table S3).

**Figure 2:**
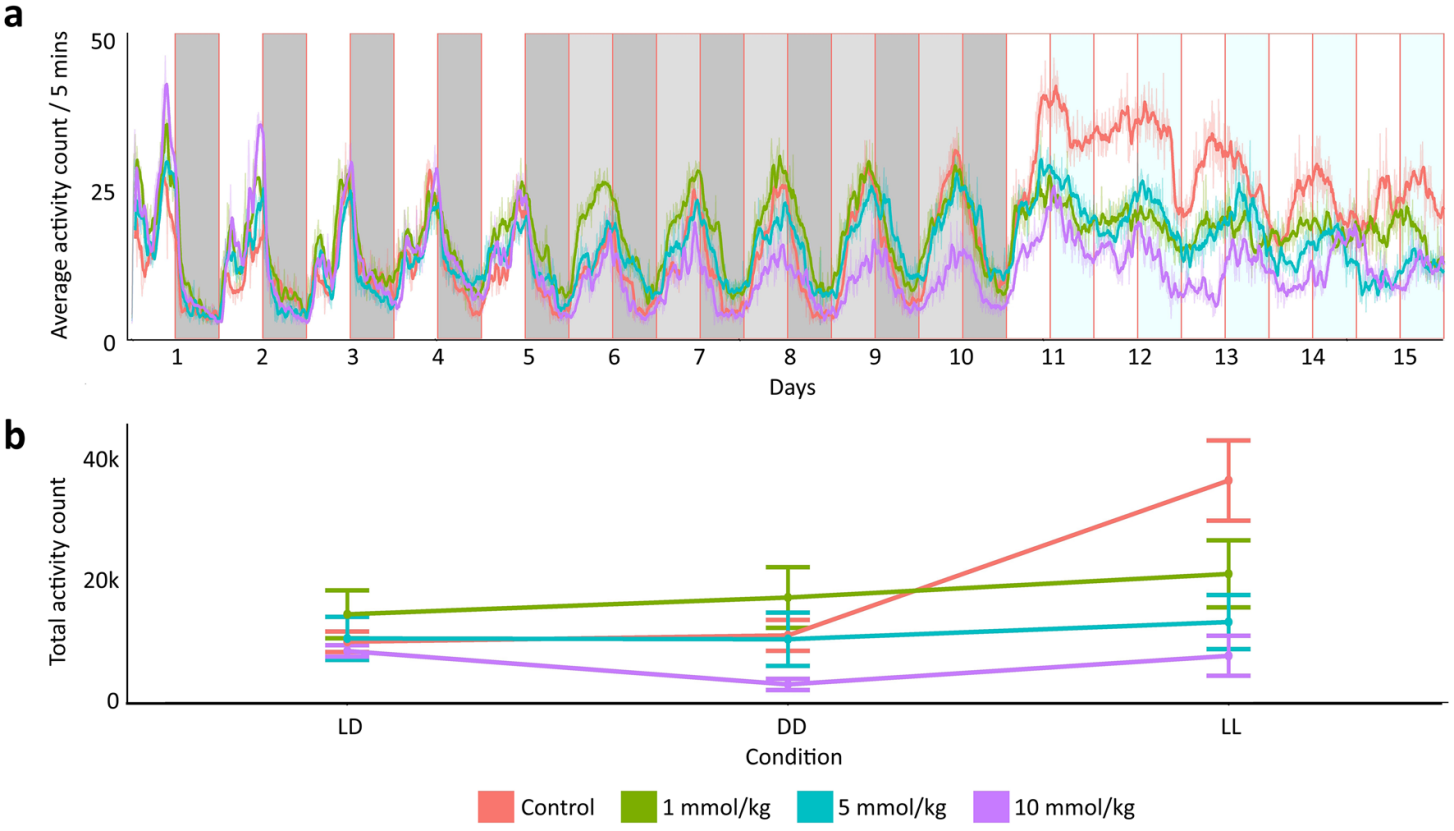
The activity graph illustrates fluctuations in LMA throughout the chronic lithium administration experiment. Dark or light grey shading indicates dark conditions, while white or light blue shading represents light conditions. These shaded areas divide the plots into 12-h intervals. A moving average filter was applied to smoothen the data, which considered 12 data points at a time as input, resulting in an average activity computed for every 60-min interval (**a**). The total activity count is depicted for LD, DD, and LL, each lasting 5 days. The data are presented as the mean of the total activity count along with the ± standard error (**b**).

Second, we compared the alteration of locomotor activities under the effect of different doses in different conditions (Fig. 2A). We used a repeated measure ANOVA test with eliminated random effects. Significant results were found for conditions (*F* (2, 34) = 8.54, *p* < 0.001), doses (*F* (3, 138) = 4.15, *p* = 0.008), and interaction effect (*F* (6, 138) = 2.69, *p* = 0.017). We applied a permutation test to our model because, according to the Shapiro–Wilk test, the distribution was not normal (*p* < 0.05). The permutation test confirmed the repeated measures ANOVA test results for conditions (B = 5000, *p* = 0.004), doses (B = 5000, *p* = 0.041), and interaction effect (B = 5000, *p* = 0.009). Then, we performed pairwise comparisons using the Wilcoxon rank-sum exact test with the Holm adjustment method. We observed no difference (*p* > 0.05) among the low and medium-dose groups across LD, DD, and LL conditions. A slight decrease was observed in the DD condition compared to the LD condition for the high-dose group (*p* < 0.05). An apparent increase was found in the control group in the LL condition. The LMA measured for the control group in the LL condition was significantly higher than all other LMA measures of all conditions and all doses (*p* < 0.05) except the low dose LMA during the LL condition (*p* > 0.05) (Supplementary Table S4, Fig. 2B) (Exact *p* values of the comparison for all pairs according to Wilcoxon test were given in supplement file, Supplementary Table S5).

**Figure 3:**
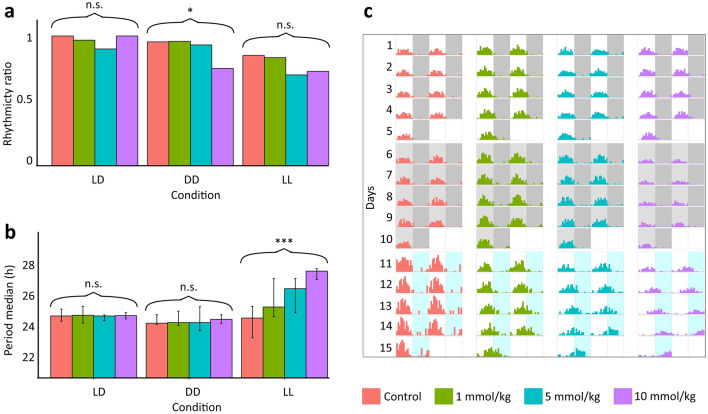
The plots represent the ratio of rhythmic individuals (**a**) and the period (in hours) of the circadian rhythm (**b**). Double-plotted actograms of representative individuals were generated using the sine wave function (Eq. 1). Dark or light grey shaded areas indicate darkness, white or light blue shaded areas indicate light periods, and the areas divide the plots into 12-h intervals (**c**). Curly brackets were used to represent the result of the logistic regression model for rhythmicity and the Spearman correlation for periodicity in the different conditions. Asterisks indicate the level of statistical significance: *p ≤ 0.05, **p ≤ 0.01, ***p ≤ 0.001, n.s. not significant. Error bars showing interquartile range.

Third, we compared the rhythmicity of circadian rhythm ratios for each condition according to the Lomb–Scargle periodogram analysis result. We found the ratios of rhythmic individuals in the LD condition for control, low, medium, and high dose groups to be 1, 0.97, 0.90, and 1, respectively. In the DD condition, 0.96, 0.96, 0.93, and 0.75. In the LL condition, 0.85, 0.83, 0.70, and 0.73, respectively (Supplementary Table S6, Fig. 3A). We inspected the association between rhythmicity and lithium treatment in each condition using the logistic regression model. We used each bee’s rhythmicity measure as a binary indicator (the value is 1 if the bee is rhythmic and 0 otherwise). We found a significant association in the DD condition (*χ*^2^ (3, 93) = 5.21, p = 0.023) but not in LD (*χ*^2^ (3, 110) = 0.001, *p* = 0.974) and LL (*χ*^2^ (3, 67) = 1.20, *p* = 0.273). Thus, rhythmicity significantly decreased in the DD condition with increasing dose. In addition, although not statistically significant, a noticeable decrease was observed in the LL condition with increasing doses of LiCl (Supplementary Table S6, Fig. 3A).

Lastly, we applied a Lomb–Scargle periodogram analysis to determine the statistics of rhythmic individuals. According to periodogram analysis, we determined the median of the periods of the circadian rhythm with an interquartile range (Q1–Q3) in hours. In the LD condition, the periods were 24.31 (23.90–24.84) for control, 24.36 (23.76–25.05) for low dose, 24.31 (23.95–24.39) for medium-dose, and 24.34 (24.08–24.57) for high dose. In the DD condition, the periods were 23.75 (23.65–24.41) for control, 23.80 (23.58–24.68) for low dose, 23.80 (23.17–25.03) for medium dose, 24.05 (23.74–24.41) for high dose. In the LL condition, the periods were 24.15 (22.62–25.05) for control, 25.00 (24.26–27.20) for low dose, 26.42 (24.56–27.20) for medium dose, and 27.77 (27.12–27.96) for high dose (Fig. 3B). Then, we determined the relationship between the periodicity and lithium treatment in each condition. We used a non-parametric Spearman correlation test because the normal distribution was not met according to the Shapiro–Wilk test (*p* < 0.05). There was no correlation in LD (*r* (106) = 0.003, *p* = 0.974), and DD (*r* (84) = 0.01, *p* = 0.908) conditions. However, a positive correlation was found in the LL condition (*r* (58) = 0.47, *p* < 0.001). Lithium affected the periodicity under the constant light condition (Supplementary Table S6, Fig. 3B). Additionally, we compared the circadian period length for each dose across conditions. According to the Kruskal–Wallis test, the light condition was not effective on the length of the circadian period for the control (*H* (2) = 0.98, *p* = 0.61) and low dose group (*H* (2) = 4.18, *p* = 0.12). Effect of the light condition on circadian period length appeared in medium (*H* (2) = 11.40, *p* < 0.001) and high dose groups (*H* (2) = 19.70, *p* < 0.001). Post-hoc Dunn test indicated the length of the circadian period in LL condition differed from LD and DD conditions for both medium (*p* = 0.002 and *p* < 0.001) and high dose (*p* < 0.001 and *p* < 0.001) groups. Thus, period length is only increased by the constant light condition for medium and high doses (Supplementary Figure S4).

Double-plotted actograms of representative individuals were generated for visualization of the LMA of each group in all conditions. They are created with the *sine wave* function (Eq. 1):

1 $$y\left(t\right)=A \ast {\mathrm{sin}} \left(2\pi f + \varphi \right)+D$$

Amplitude (*A*), center amplitude (*D*), and frequency (*f*) variables were tuned according to the LMA averages and lengths of the circadian period of each dose group in each condition, also, arrhythmicities were added as noise (Fig. 3C).

## Discussion

The principal finding of our study was that lithium altered honey bees' LMA and circadian rhythms as predicted by the suppression of induced activity and regulation of circadian activity hypotheses. According to the acute lithium administration experiment results, lithium treatment lowered the light-induced high activity in honey bees yet did not affect the low activity observed under dark conditions (Fig. 1). Similarly, in the chronic experiment, lithium decreased the elevated locomotor activity in the constant light condition. (Fig. 2). We also examined the effect of lithium on rhythmicity and the circadian period in the chronic lithium administration experiment. The lithium treatment disrupted rhythmicity significantly by lowering the rhythmicity ratio in the DD condition. Additionally, we observed a decreasing trend in the rhythmicity ratio under the LL condition. The circadian period was not affected by different light conditions in the control group, yet it lengthened with lithium treatment under the constant light condition.

In our study, constant light triggered a higher activity, as reported before^27^. We likened this to mania-like behavior. In other bipolar disorder models, different methods were applied to increase the activity to develop a mania-like state: paradoxical sleep deprivation^40^, drugs^41^, and mutations in the *Clk* gene, which is important for circadian rhythms^42^ have been used to trigger mania-like activity. Lithium decreased the elevated activity in all three studies^40–42^. In our study, similar to previous studies, lithium treatment reduced bees’ elevated activity under acute and chronic light conditions when compared to control. In contrast, control and lithium-treated bees exhibited similar, lower locomotor activity under dark conditions. This observation is consistent with lithium maintenance trials in patients with bipolar disorder, where lithium is primarily effective against mania^43–45^.

In addition to effects on locomotor activity levels, lithium also impacts the strength of circadian rhythms and the length of the circadian period. The lithium studies have shown that lower rhythmicity was observed in fruit flies in DD^14^ and house flies (*Musca domestica*) under low light^32^. In our study, we also observed the disruptive effect of lithium on bees’ rhythmicity. The possible molecular mechanism underlying our observations may also be attributed to lithium inhibition of the activity of vertebrate *Gsk-3* ortholog *Sgg*, which is related to regulating signal transduction, xenobiotic stress resistance, and neuronal health as it was proposed for fruit flies^17,46^.

Results of long-term experiments on diurnal vertebrates such as goldfish (*Carassius auratus*), and squirrel monkeys (*Saimiri sciureus*) have found a lengthening of the circadian period with lithium treatment, and these were measured in the LL condition^33,34^. In our study, we observed an increase in the circadian period only in the LL condition. In contrast to our study, the period has been shown to lengthen under the DD condition in other insects subjected to lithium treatment. In previous investigations, lithium has been shown to affect the circadian period length in various organisms, including nocturnal mammals such as mice and hamsters (*Mesocricetus auratus*), as well as insects such as cockroaches (*Leucophaea maderae*), fruit flies, and house flies, under DD or weak red light conditions^14,32,35–37^.

The lengthening of the circadian period under constant light in insects is widely accepted (Aschoff rule)^47^. Surprisingly, we did not observe a statistically significant increase in the period under the LL condition (24.15 h) when compared to the DD condition (23.8 h) in the control group. Our results suggest that honey bees may differ from other insects in their circadian period regulation. Honey bees carry the mammalian-type *Cryptochrome* gene *Cry2* but lack the *Cry1* and *Tim1* genes, which have been shown to regulate circadian rhythm in other insects^48^. The absence of these interactions in honey bees may be why we did not observe a lengthening in the circadian period in DD condition with lithium treatment. However, instead of light-sensitive *Cry1*, vertebrate-like opsin called pteropsin was found to be predominant in honey bees^16^.

In fact, similar to bees, *Cry1* loss-of-activity mutant fruit flies did not exhibit period lengthening in the LL conditions^49^. The absence of *Cry1* in honey bees may explain why we did not observe period lengthening in the control group under constant light conditions (LL). The inactivation of *Sgg* was reported to cause circadian period lengthening under DD conditions^18^, and *Sgg* was also found to regulate the *Cry1* and *Tim1* genes in fruit flies^41^. Therefore, we propose that, as observed in diurnal vertebrates, the interactions between SGG and pteropsin might cause the circadian period lengthening with lithium treatment under the LL condition in bees.

As a caveat, the results of any study on bees in isolation may be confounded by the absence of social interactions. However, using control groups under similar isolation conditions leads to useful mechanistic insights in honey bee studies. For instance, research has shed light on the development of circadian rhythms^50^, the role of temperature in training the circadian clock^51^, and the toxicity of particular compounds^30,31^. Our study employed the same LMA monitoring device that had demonstrated reliability in previous research^30,51^. In our acute lithium administration experiment, the duration of social isolation was only one day. Under different light conditions, controls and salt treatment groups exhibited similar LMA to each other and differed from the lithium treatment. We conclude that increased LMA under constant light and its suppression by lithium is independent of any potential social isolation effects. In addition, the outcome of the chronic lithium administration experiment (15-day social isolation) is consistent with the outcome of the acute administration (1-day social isolation), even though the social isolation periods were very different. In both experiments, we observed an increase in LMA under constant light. Additionally, upon the start of the LL condition in the chronic experiment, a sharp increase in LMA was observed (Fig. 2A). We inferred that LMA was affected by constant light and lithium, and the effects were not related to social stress.

Our study also provides data on the safety of the recommended acaricide dose of LiCl. A significant increase in the mortality of *Varroa* was observed in the group of bees that were individually fed 10 µl sucrose solution with 25 mM LiCl^1^. Even though our low dose (50 mM) in acute lithium administration experiments was twice the effective dose on *Varroa*, it did not affect LMA. In our chronic lithium administration experiments, even at the highest dose (10 mmol/kg corresponds to about 70 ppm), there was no observable effect either in LMA or in the rhythmicity and periodicity of the circadian rhythm for the first five days under the LD condition. Our highest dose in the chronic lithium administration experiment was 3.5 times the effective dose for managing *Varroa* (25 mM LiCl in 1:1 sucrose syrup corresponds to 19.88 ppm), and it was administered to the bees ad libitum. Thus, our results support the suggested safe LiCl dose to combat *Varroa*. Although we had conclusive data on LMA and circadian rhythm in adult bees there is still a need to further determine the effect of lithium on bee behavior. For future research on honey bees, it will be important to consider the unique circadian rhythm ontogeny of bees, driven by age and social environment. In honey bees, the rhythmicity development depends on the worker bees' roles in the colony. Young nurse bees do not display circadian rhythms when in contact with the brood. In contrast, older forager bees exhibit robust circadian oscillations in the expression of circadian-related genes such as *Per*, *Cry2*, *Tim2*, and *Clk*^50^.

In conclusion, this research highlights that lithium's impact on honey bees may have significant implications for bee health. The safe acaricidal use of lithium is especially important for the long-term sustainability of global food production. Lastly, based on our findings and previous reports by other researchers, we propose the honey bee as an experimental animal model for lithium effects in bipolar disorder due to similarities between bees and humans in related responses, genes, and behavior^23–26^.

## Material and method

### Sampling

Honey bee (*Apis mellifera*) hives in the apiary of Ankara University's Veterinary Faculty sources were used in this study. Hive entrances were blocked with plastic wire meshes. Returning forager bees that could not enter the hive congregated on the wire meshes and were collected into small containers^52^. Samples were from at least three hives and pooled for each analysis to mitigate the colony effect.

Although scientific experiments on honey bees in Türkiye are not subject to specific regulations, our study was conducted with the commitment to minimize the harm and ethical concerns.

### Acute experiments

We observed a mortality rate of around %50 in the group of bees fed one time 10 μl sucrose solution with 450 mM LiCl after 24 h. Then, we determined the highest dose and adjusted other doses by reducing them by one-third of the highest dose. Sucrose solution (50% w/v) containing 50, 150, and 450 mM LiCl for treatments and a pure sucrose solution (50% w/v) for the control group were prepared at room temperature. In addition, we used the 450 mM NaCl treatment as a control for the hyper-osmotic stress that the 450 mM salt solution may cause. Bees were fed individually 10 μl of solutions. The 10 μl solution was dropped into a small-sized stainless steel laboratory spoon via a micropipette. A cotton swab dipped in the sucrose solution was touched on the honey bee’s antenna for the bee to elongate its proboscis. Then, we fed the solution that was found inside the laboratory spoon. We made sure that the bee drank all the solution.

The LMA experiment was performed as described in previous studies^30,51^. After treatments, honey bees of experimental groups were individually transferred into perforated 15 ml falcon tubes. A piece of fondant sugar was placed into the cap of each tube as food and covered with two layers of cheesecloth to prevent the sticking of bees to the fondant sugar. The LMA monitoring device produced by Trikinetics Inc (TriKinetics Inc, Waltham, MA, USA) was used in our study. We used 4 modules with 24 holes to hold a single 15 ml falcon tube. Each falcon tube includes a single bee. Thus, the sample size for each experiment group was 24. The three infrared sensors around each hole gave a positive signal when the bee in the Falcon tube moved through the section with the beams^53^. An environmental monitor was also attached to the system, constantly measuring and recording temperature, light, and humidity levels. The LMA monitoring modules were incubated at 33 °C and 65% humidity.

The LMAs of bees in treatment and control groups were measured for 24 h in two experiments, one in constant darkness and another in a constant light environment.

### Chronic experiments

The chronic LMA experiment was performed according to previous studies^30,51^. LiCl was delivered to the bees in a piece of custom-made bee candy. The bee candy was placed into the cap of each perforated 15 ml falcon tube and covered with two sheets of cheesecloth. Bee candy consisted of ten parts of honey, 54 parts of powdered sugar, and two parts of distilled water in grams. Designated amounts of LiCl were dissolved in the water fraction of treatment groups (1, 5, and 10 mmol/kg of LiCl). Collected forager honey bees were put into falcon tubes individually. For each group, 32 bees were used. The falcon tubes were put into the modules of the LMA monitoring device. A water system was assembled into the modules of the LMA monitoring device. The water system consisted of 30-cm PVC (polyvinyl chloride) pipes filled with water. The plastic straws inserted into these pipes entered the falcon tubes’ rear end. The water was delivered to the bees through the filter papers placed in the plastic straws. Previous studies also used the same system^30,51^. The chronic LMA experiment was performed under the same conditions (33 °C, 65% humidity) for 15 days with 5 days in a 12-h light and dark cycle (light:dark, LD) followed by 5 days in constant darkness (dark:dark, DD) and the final 5 days under continuous illumination (light:light, LL).

### Statistics

All statistical analyses were conducted in RStudio with the R 4.3 version^54^.

The normality of the sample distribution was checked with the Shapiro–Wilk test.

In acute experiments, in the case of the distributions that did not follow the normal distribution, the activity differences across the groups were compared by the non-parametric Kruskal–Wallis test followed by a post-hoc Dunn test. The relationship between the dosages and LMA was examined through a regression analysis. We checked group differences in mortality via Chi-Square analysis.

In chronic treatment experiments, a survival analysis was done by log-rank test to compare mortality differences. Alteration of activities under the effect of different lithium doses in different light exposure conditions was examined via repeated measure ANOVA tests with eliminated random effects. Then, pairwise comparisons were applied using the Wilcoxon rank-sum exact test with the Holm adjustment method. Also, a permutation test was used to verify the repeated measure ANOVA test results. Circadian rhythm analysis determining the periodicity and rhythmicity was conducted with a set of R packages called "Rethomics", available at https://rethomics.github.io^55^. The Lomb–Scargle periodogram analysis was used to determine the rhythmic individuals and lengths of the circadian periods. Then, the logistic regression model was used to determine the association between rhythmicity and lithium treatment. The Spearman correlation test was performed to determine the association between the circadian period and lithium treatment for each condition.

### Supplementary Information

Supplementary Information.

## Data Availability

The datasets generated during and/or analyzed during the current study are available in the Zenodo repository, https://doi.org/10.5281/zenodo.7939046.
