# Supplementary material for: Effects of lithium on locomotor activity and circadian rhythm of honey bees
Source: Sci Rep. 2023 Nov 13;13:19861. doi: 10.1038/s41598-023-46777-7 (PMC10646147; doi:10.1038/s41598-023-46777-7)
Supplement: Supplementary file 1 — Supplementary Information. [file 41598_2023_46777_MOESM1_ESM.pdf]

Effects of Lithium on Locomotor Activity and Circadian Rhythm of Honey Bees

Babur Erdem[1, 2 *], Okan Can Arslan[2], Sedat Sevin[3], Ayse Gul Gozen[1], Jose L. Agosto[4], Tugrul Giray[4], Hande Alemdar[5]

1. Department of Biological Sciences, Middle East Technical University, Ankara, Türkiye
2. Center for Robotics and Artificial Intelligence (ROMER), Middle East Technical University, Ankara, Türkiye
3. Department of Pharmacology and Toxicology, Faculty of Veterinary Medicine, Ankara University, Ankara, Türkiye
4. Department of Biology, University of Puerto Rico, Rio Piedras, Puerto Rico
5. Department of Computer Engineering, Middle East Technical University, Ankara, Türkiye

***** Babur Erdem: ebabur@metu.edu.tr

Acute experiments

Table S1. Descriptive statistics for acute LMA experiments. Sample sizes (n), mean of the total activity count for 24 hours, standard deviations (Std. Dev.), and standard errors (Std. Err.) are given in the table.

		n	Mean	Std. Dev.	Median	Std. Err.
Dark Environment Experiments	Control	24	2220.46	1596.29	1913.00	325.84
	50 mM LiCl	23	2070.65	1322.75	1721.00	275.81
	150 mM LiCl	22	3184.46	1932.33	2422.50	411.97
	450 mM LiCl	25	2259.08	1829.77	1748.00	365.95
	450 mM NaCl	24	3196.58	2364.28	2480.00	482.61
Light Environment Experiment	Control	22	4908.55	3246.16	3827.50	692.08
	50 mM LiCl	24	3360.38	1739.24	3134.50	355.02
	150 mM LiCl	20	2695.30	1694.88	2509.50	378.99
	450 mM LiCl	20	2426.75	1373.42	2477.00	307.11
	450 mM NaCl	24	4259.17	1824.97	3728.00	372.52

Table S2. Mortality ratios of the groups acute LMA experiments in the dark and light environment.

Experiment	Treatment	Dose	Mortality Ratio
Acute LMA Experiment in Dark Environment	LiCl	Control	0.00
	LiCl	50 mM	0.04
	LiCl	150 mM	0.08
	LiCl	450 mM	0.48
	NaCl	450 mM	0.00
Acute LMA Experiment in Light Environment	LiCl	Control	0.08
	LiCl	50 mM	0.00
	LiCl	150 mM	0.17
	LiCl	450 mM	0.17
	NaCl	450 mM	0.00

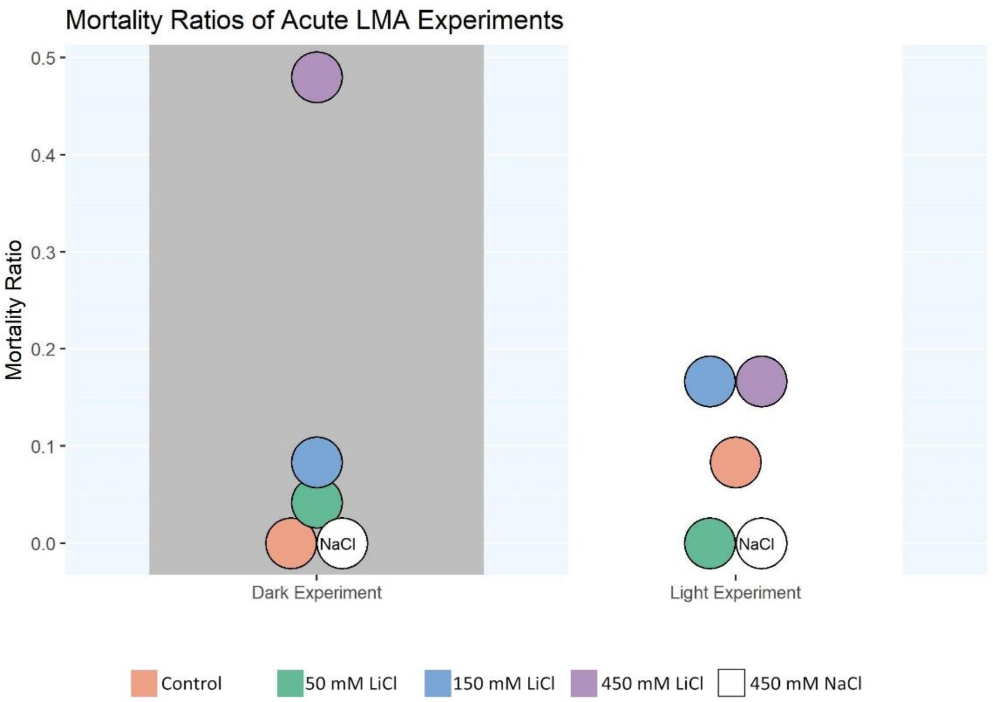

Figure S1. Mortality ratios of the groups' acute LMA experiments in the dark and light environment.

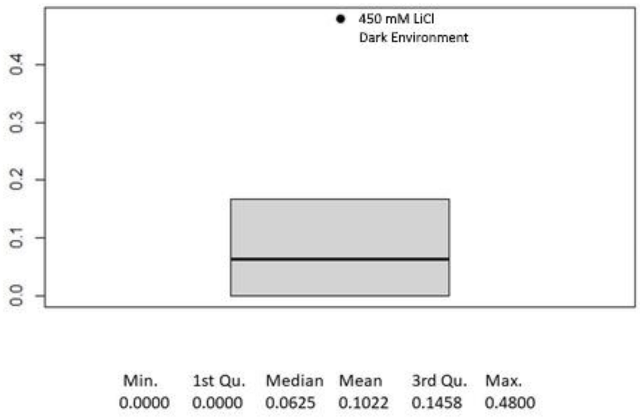

Figure S2. Box plot and descriptive statistics of mortality ratios of all dose groups in both dark and light experiments. The death ratio of the 0.45 M group in the dark experiment is 0.48. The high-dose group in the dark environment appeared as an outlier.

Chronic experiments

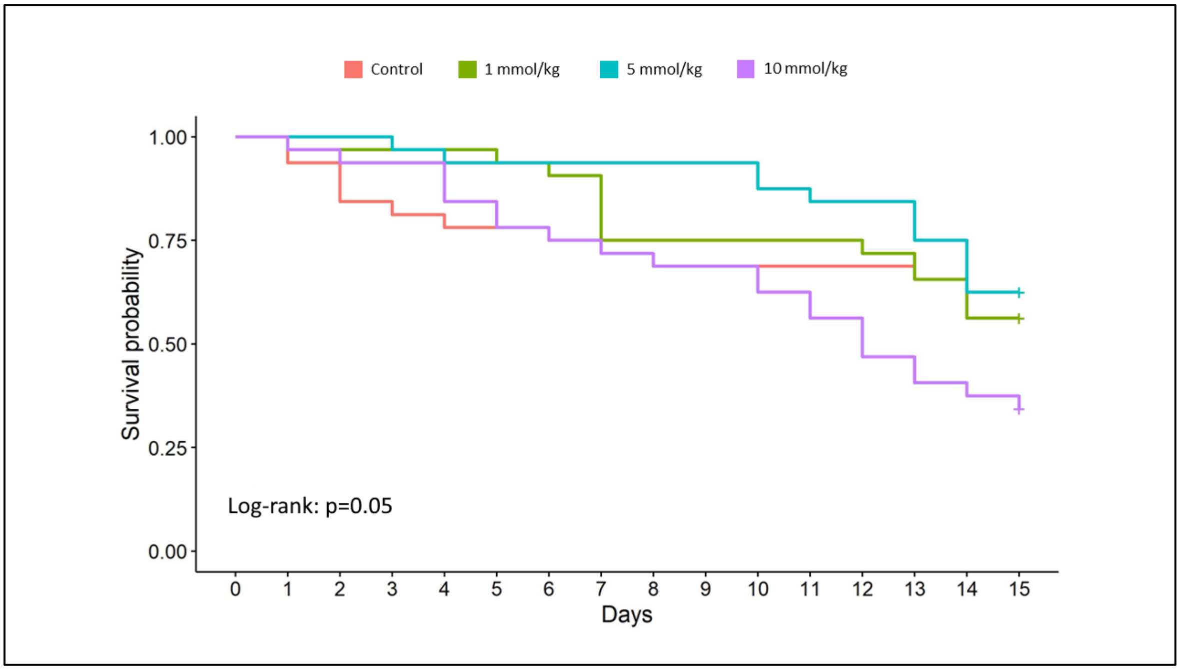

Figure S3. Log-rank test plot. There was no difference found between the groups ($\chi 2 = 7.9$, p = 0.05)

Table S3. Pairwise comparisons of the survival probabilities of the doses using Log-Rank test. P values are adjusted with BH method

	**Control**	**1mmol/kg**	**5mmol/kg**	**10mmol/kg**
**Control**	-	0.871	0.821	0.138
**1mmol/kg**	0.871	-	0.751	0.138
**5mmol/kg**	0.821	0.751	-	0.051
**10mmol/kg**	0.138	0.138	0.051	-

Table S4. Descriptive statistics for chronic LMA experiments. Sample sizes (n), mean of the total activity count in conditions as LD, DD, and LL, (total activity count in 5 days), standard deviations (Std. Dev.), and medians are given in the table.

	Condition	n	Mean	St. Dev.	Median
Control	LD	20	12445.80	6994.88	11653.00
	DD	20	13415.50	10570.17	11588.50
	LL	20	37158.90	27481.49	25793.00
1 mmol/kg	DD	18	19215.06	19739.43	9329.50
	LD	18	16655.72	15558.48	12473.50
	LL	18	22845.72	21835.67	10716.00
5 mmol/kg	LD	20	12952.80	14754.55	9651.50
	DD	20	12826.55	18271.93	7354.50
	LL	20	15472.85	18533.57	9632.50
10 mmol/kg	LD	11	11028.18	2903.24	11207.00
	DD	11	5893.27	2844.56	5100.00
	LL	11	10289.27	10167.60	5496.00

Table S5. Pairwise comparisons of LMA levels in chronic experiment for all data points. The comparisons were achieved using the Wilcoxon rank sum exact test, and p values were adjusted with the Bonferroni method. In the table, High, Med., Low, and Cont. indicates 10 mmol/kg, 5 mmol/kg, 1 mmol/kg, and control, respectively. LD: Light/Dark, DD: constant dark, LL constant dark.

	Cont_LD	Cont_DD	Cont_LL	Low_LD	Low_DD	Low_LL	Med_LD
Cont_DD	0.93966	-	-	-	-	-	-
Cont_LL	0.00114	0.00189	-	-	-	-	-
Low_LD	0.87670	0.57117	0.00725	-	-	-	-
Low_DD	0.98848	0.87670	0.03207	0.84468	-	-	-
Low_LL	0.78234	0.52650	0.12355	0.93014	0.78234	-	-
Med_LD	0.53153	0.84468	0.00064	0.39134	0.93014	0.78234	-
Med_DD	0.27354	0.39134	0.00064	0.10341	0.41790	0.22031	0.45197
Med_LL	0.74179	0.86015	0.00438	0.41790	0.78234	0.41790	0.87670
High_LD	0.78234	0.93966	0.00189	0.78234	0.93966	0.92661	0.60251
High_DD	0.00725	0.02558	3e-05	0.00189	0.09429	0.03065	0.02558
High_LL	0.38889	0.38889	0.00466	0.11174	0.35650	0.10798	0.28666

	Med_DD	Med_LL	High_LD	High_DD
Cont_DD	-	-	-	-
Cont_LL	-	-	-	-
Low_LD	-	-	-	-
Low_DD	-	-	-	-
Low_LL	-	-	-	-
Med_LD	-	-	-	-
Med_DD	-	-	-	-
Med_LL	0.60251	-	-	-
High_LD	0.35650	0.85489	-	-
High_DD	0.27354	0.11174	0.00505	-
High_LL	0.86015	0.61916	0.35650	0.78465

Table S6. Sample sizes (n), number of rhythmic individuals in the samples, the ratio of the rhythmic individuals to the sample, and descriptive statistics for the period (in hours) of the circadian rhythm as mean, standard deviation, and median are given in the table. In addition, the results of the Spearman correlation test for periodicity, and logistic regression test for rhythmicity are included in the table.

		n	*n* (rhythmic)	Rhythmic ratio	Period Mean	Period St.Dev.	Period Median	Spearman correlation		Logistic regression	
								r	*p*	*χ2*	*p*
LD	Control	25	25	1	24.10	1.45	24.31	0.003	.97	0.001	.97
	1 mmol/kg	31	30	0.97	24.53	1.04	24.36				
	5 mmol/kg	30	27	0.90	24.21	0.64	24.31				
	10 mmol/kg	26	26	1	24.45	0.95	24.34				
DD	Control	22	21	0.96	24.09	1.04	23.75	0.01	.91	5.21	.02
	1 mmol/kg	24	23	0.96	24.03	0.75	23.80				
	5 mmol/kg	29	27	0.93	24.41	1.92	23.80				
	10 mmol/kg	20	15	0.75	24.06	1.15	24.05				
LL	Control	20	17	0.85	23.77	3.09	24.15	0.47	<.001	1.20	.27
	1 mmol/kg	18	15	0.83	25.20	3.73	25.00				
	5 mmol/kg	20	14	0.70	26.04	2.02	26.42				
	10 mmol/kg	11	8	0.73	27.74	1.43	27.77				

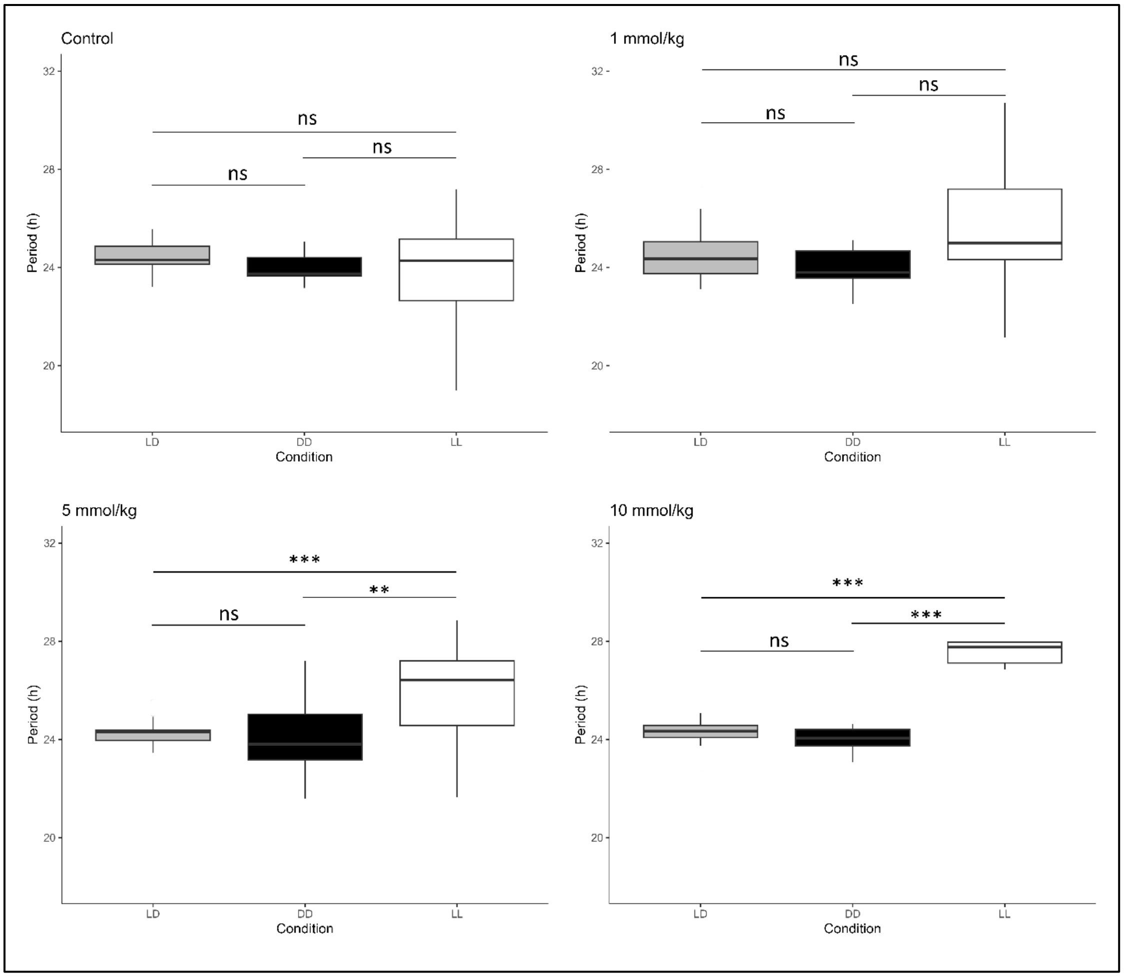

Figure S4. Light conditions did effective on the circadian period for medium and high dose ($p < .001$) but not for control and low dose groups ($p > .05$) according to the Kruskal-Wallis test. The length of the circadian period in LL condition was significantly higher than in other conditions according to the Dunn test, * $p < .05$, ** $p < .01$, *** $p < .001$, ns: not significant. Grey boxes represent LD, black boxes are DD, and whites are LL.
